# Recorded interviews with human and medical geneticists

**DOI:** 10.1007/s00439-016-1744-9

**Published:** 2016-11-15

**Authors:** Peter S. Harper

**Affiliations:** Cardiff University, Cardiff, UK

## Abstract

A series of 100 recorded interviews with human and medical geneticists has been carried out and some general results are reported here. Twenty countries across the world are represented, mostly European, with a particular emphasis on the United Kingdom. A priority was given to older workers, many of whom were key founders of human genetics in their own countries and areas of work, and over 20 of whom are now no longer living. The interviews also give valuable information on the previous generation of workers, as teachers and mentors of the interviewees, thus extending the coverage of human genetics back to the 1930s or even earlier. A number of prominent themes emerge from the interview series; notably the beginnings of human cytogenetics from the late 1950s, the development of medical genetics research and its clinical applications in the 1960s and 1970s, and more recently the beginnings and rapid growth of human molecular genetics. The interviews provide vivid personal portraits of those involved, and also show the effects of social and political issues, notably those arising from World War 2 and its aftermath, which affected not only the individuals involved but also broader developments in human genetics, such as research related to risks of irradiation. While this series has made a start in the oral history of this important field, extension and further development of the work is urgently needed to give a fuller picture of how human genetics has developed.

## Introduction

Human genetics and the overlapping field of medical genetics have been prominent themes for research since the beginnings of modern genetics just over a century ago and even before this, some of the earliest observations and investigations on the mechanisms of heredity being based on human characteristics and inherited disorders. The differentiation of these areas of genetics into a specific scientific discipline (human genetics) and a distinct medical speciality (medical genetics), however, has only become marked over the past 70 years, with the beginning of human genetics dating essentially from the end of World War 2 and the birth of medical genetics occurring around a decade later (Harper 2008).

These relatively recent origins mean that many founders of the field and other early workers are still living, thus giving the opportunity of recording memories of their lives and experiences through interviews. Such interviews are particularly relevant in documenting the history of some developments made possible by major technological advances, such as human chromosome research, which began largely in the mid-1950s and also for recording the practical applications of these discoveries, which have formed a large part of medical genetics, essentially beginning around 1960, and which are now playing an increasing role in wider clinical and laboratory medicine.

Despite these opportunities, and despite the importance of human and medical genetics for both science and medicine overall, little interest in human and medical genetics, whether for oral history or for written documentation, has been taken by historians of science and medicine until very recently, while those actually working in the area have also been slow in attempting to document the field, or even to record their own specific experiences. This contrasts with the considerable historical studies of eugenics in the early 20th century (Kevles 1985), and of the founders of molecular biology in the mid-part of the century (Judson 1979; Olby 1974) and also with historical interest in the recently completed Human Genome Project at the end of the 20th century.

In 2002 the *Genetics and Medicine Historical Network* (http://www.genmedhist.org) was founded to correct this major deficiency, with one of the primary aims being to undertake a series of recorded interviews with human and medical geneticists. The present paper documents the key features of a series of 100 recorded interviews (Table 1), almost all of which are now accessible in edited form on the Web, with the original material archived and available to scholars.

**Table 1:** 100 recorded interviews with human geneticists

Aymé, Ségolène (Marseille; Paris) [85]	de la Chapelle, Albert (Helsinki; Columbus, Ohio) [84]
Bakker, Bert (Egbert) (Leiden) [77]	Delhanty, Joy (London) [25]
Baraitser, Michael (London) [33]	Donnai, Dian (Manchester) [63]
Bates, Gill (London) [57]	Edwards, Anthony (Cambridge) [29]
Berg, Kåre (Oslo) [49]^a^	Edwards, John (Oxford; Birmingham UK) [14]^a,b^
Berger, Roland (Paris) [38]^a^	Eiberg, Hans (Copenhagen) [95]
Berry, Caroline (London) [20]	Emery, Alan (Manchester; Edinburgh) [48]
Berry, RJ (Sam) (London) [31]	Evans, H John (Edinburgh) [04]^a,b^
Bertram, Ewart (Mike), jointly with Moore, Keith (London, Ontario; Toronto) [23]^b^	Evans, Edward (Harwell) [15]^b^
	Farndon, Peter (Birmingham, UK) [97]
Bobrow, Martin (Oxford; London; Cambridge) [24]	Feingold, Josué (Paris) [35]
Bochkov, Nikolai (Moscow) [46]	Ferguson-Smith, Malcolm (Glasgow; Cambridge) [03]^b^
Bodmer, Walter (Oxford) [68]	Frézal, Jean (Paris) [44]^a^
Boué, André and Joelle (Paris) [43]^a^	Fraccaro, Marco (Pavia) [09]^a,b^
Brøgge, Anton and van der Hagen, CB (Oslo) [50]	Fraser, George (Oxford) [32]
Brunner, Han (Nijmegen) [99]	Fryns, Jean-Pierre (Leuven) [65]
Burn, John (Newcastle) [100]	Galjaard, Hans (Rotterdam) [76]
Burns, Joan (Madison) [53]	Gilgenkrantz, Simone (Nancy) [39]
Clarke, Angus (Cardiff, Newcastle) [96]	Ginter, Yevgeny (Moscow) [47]
Conneally, Michael (Indianapolis) [22]	Goodfellow, Peter (London; Cambridge) [98]
Crow, James (Madison) [54]^a^	Gusella, James jointly with MacDonald, Marcy (Boston, USA) [82]
Dausset, Jean (Paris) [41]^a^	Haan, Eric (Adelaide; Melbourne) [86]
Davies, Kay (Oxford, London) [80]	Hamerton, John (London; Winnipeg) [21]^a,b^
Harnden, David (Edinburgh; Manchester) [08]^b^	Modell, Bernadette (London) [70]
Harper, Peter (Cardiff) (interviewed by Angus Clarke) [16]	Mohr, Jan (Oslo; Copenhagen) [51]^a^
	Morton, Newton (Honolulu; Southampton) [34]
Harris, Henry (Oxford) [67]^a^	Munnich, Arnold (Paris) [93]
Harris, Rodney (Manchester) [59]	Nevin, Norman (Belfast) [26]^a^
Hastie, Nick (Edinburgh) [11]	Pembrey, Marcus (London) [62]
Hopkinson, David (London) [81]	Polani, Paul (London) [01]^a,b^
Hughes, Helen (Cardiff; Toronto) [90]	Povey, Sue (London) [71]
Hulten, Maj (Stockholm, Sweden; Birmingham, UK) [10]^b^	Réthoré, Marie-Odile (Paris) [37]
	Read, Andrew (Manchester) [64]
Jacobs, Patricia (Edinburgh; Honolulu; Salisbury) [06]^b^	Roberts, Derek (Newcastle) [02]
Jeffreys, Alec (Leicester) [75]	Romeo, Giovanni (Genoa; Bologna) [73]
Jenkins, Trefor (Johannesburg) [69]	Sampson, Julian (Cardiff, Glasgow) [91]
Johnston, Alan (Aberdeen) [74]^a^	Scriver, Charles (Montreal) [56]
Kaplan, Jean-Claude (Paris) [40]	Searle, Anthony (Harwell) [19]
Lam, Stephen (Hong Kong) [87]	Sequeiros, Jorge (Porto) [88]
Laurence, K Michael (Cardiff, Bern) [13]	Snell, Russell (Cardiff; Auckland) [83]
Laxova, Renata (Brno; Madison) [55]	Sutherland, Grant (Adelaide) [60]
Laziuk, G (Minsk) [45]	Tobin, Allan (Los Angeles) [52]
Lee, Muriel (Edinburgh) [12]^b^	Turleau, Catherine (Paris) [42]
Lindsten, Jan (Stockholm) [27]^b^	van Ommen, GertJan (Leiden) [78]
Lyon, Mary (Harwell)^a^ [18]	vanden Berghe, Herman (Leuven) [66]
Macek, Milan (Sr) (Prague) [89]	Vogel, Friedrich (Heidelberg) [05]^a^
Mandel, Jean-Louis (Strasbourg) [72]	Warburg, Mette (Copenhagen) [17]
Maroteaux, Pierre (Paris) [36]	Weatherall, David (Liverpool; Oxford)[30]
Mattei, Jean-François (Marseille) [94]	Went, Loe (Leiden) [79]
Medvedev, Zhores (Obninsk, Russia; London) [58]	Williamson, Robert (Glasgow; London; Melbourne) [61]
Mitelman, Felix (Lund) [92]	Zech, Lore (Stockholm) [28]^a^
Mittwoch, Ursula (London) [07]	

Complete list of interviews by P. S. Harper 2003–2014

[–] Indicates chronological sequence of interviews

^a^Deceased

^b^Audioclip of recording available on http://www.genmedhist.org website

Although material from the interview series has been used for a range of presentations and in two books (Harper 2006a, 2008), no detailed description of the project and of the interview series as a whole has been made until now, apart from a short note in the *Genmedhist* Newsletter (Harper 2011).

## Aims

The main factor initiating the interview series was recognition that the founders of human and medical genetics were now elderly and often frail, in some cases already deceased, and that there was no systematic initiative being undertaken to record their memories and experiences, particularly across Europe. The primary aim was thus to provide a permanent record for as many as possible of the workers who had made prominent contributions to the field. In the light of a comparable series for American human geneticists (http://ohhgp.pendari.com/collection), initiated around the same time but since discontinued, a conscious attempt was made for the two series to be complementary, rather than to duplicate each other, with the initiative described here focusing primarily on European workers.

A second aim was to make the material as widely available as possible, leading to interview transcripts being placed on the Web and forming part of the *Genetics and Medicine Historical Network* website (http://www.genmedhist.org/interviews) as noted above, rather than waiting for completion of the entire project. Since the development of human and medical genetics is a topic of widespread general interest, not limited to geneticists or historians, it was decided to make no restrictions on access to the Web based interviews, provided that specific permission had been given by interviewees.

Finally, it was hoped that archiving of the full material (see below), including the unedited primary transcripts and the recordings themselves, along with correspondence and other ancillary material, would allow more extensive study by scholars, and provide the foundation for later detailed historical analysis, including the possibility of further, more specific interviews with those in the series.

## Methods

Although technical and operational aspects of the interview series may seem trivial to geneticists, and possibly self-evident to historians and social scientists, they are given briefly here, since it is hoped that others who have no previous interviewing experience will also wish to interview colleagues in their particular field of genetics. The following points may be relevant.

1. All interviews in the first phase of the work (2003–2008) used a Sony minidisc recorder with additional recording microphone, which proved compact, easily portable, unobtrusive, and gave high quality recordings each lasting up to 4 h. The minidiscs were labelled at the time of interview with full name and date—as if they were a laboratory sample—to avoid future confusion. For the latter part of the series (2010–2014) a Sony digital recorder was used, with the advantage of recordings being directly transferable to computer. Previous minidisc recordings were also transferred to computer, an archival file and also one for use in corrections and editing being created.
2. English was the language used for most interviews, though some with French workers were carried out completely or partly in French, translation being made by the author with help from the Cardiff University translation service. Not all interviewees were fully comfortable with spoken English, but they, as well as native English speakers, were discouraged from trying to improve grammar and style during the process of correcting and editing the interview transcripts.
3. A ‘background note’ was written or dictated immediately or as soon as possible after completion of the interview, giving information on general aspects of the interview, technical or other problems encountered and on topics that were relevant but not included in the recording. Informal discussions often continued for a considerable time after the end of the formal interview and interviewees sometimes used this as an opportunity to raise especially sensitive or controversial topics.
4. A primary transcript was made (see “Acknowledgments”), and then corrected by the author while listening to the recording; this was then sent to the interviewee for further correction and editing, often a prolonged process involving several iterations. Where the edited version was likely to differ substantially from the original (only a few instances), two versions were sent—edited and unedited.
5. All recorded interviews were made by one individual (the author), with the exception of the interview with the author himself.
6. Written permission of a general nature (necessary for copyright reasons) was obtained, at the time of interview whenever possible, but specific permission for the edited material to be placed on the Web was requested later, at the time of correcting the transcript.
7. Preparation for the Web. A simple structure was created for the interview series which allowed each interview transcript to be placed as a PDF on a page which also gave basic details of the interviewee, including a photograph and brief biography. So far, only a small number of excerpts from the audio files are available on the Web (indicated in Table 1). These were originally published as a CD forming part of an earlier book on the beginnings of human cytogenetics (Harper 2006a).

## Interview structure

No rigid structure was employed, but for most interviews a biographical sequence was used. The duration was usually between 1 and 2 h, considerably less than most history of science or medicine interviews conducted by historians. In general more time was spent on a worker’s early life and career, rather than on later years, especially for those whose later career involved running a large institute or department. Personal life (apart from childhood and early years) was not directly inquired about unless of relevance to the person’s work, though these aspects were often volunteered, and in many cases proved of considerable interest and importance. All interviewees were asked two specific questions at the conclusion of the interview: Which piece of work or other contribution in their career did they value most and who did they regard as the principal influences and mentors in their life and career?

## The interview series

Interviews started in November 2003, and continued until late 2008, by which time a total of 72 recorded interviews had been made. New interviews were then halted to allow time for editing and Web preparation, being resumed in 2010, after the original series had been established on the http://www.genmedhist.org website. The series was concluded in late 2014 after 100 interviews had been completed. The number of interviewees totalled 104, since there were four joint interviews.

*Geographical distribution* by country is given in Table 2. As already explained, no attempt was made to cover America systematically, this being partly because of the parallel American series then in progress, (ohhgp.pendari.co/collection), which unfortunately has since been discontinued; partly because the travel and other expenses would have required considerable financial support, which was not available. The UK predominance results partly from logistic reasons determining easy access, but also because an attempt was made to interview a wide range of less well-known, though still important scientists and clinical geneticists, something not feasible for other European countries on account of time and cost constraints.

**Table 2:** Countries represented in the interview series

Country	Number interviewed
Australia	2
Belarus	1
Belgium	2
Canada	4
Czech Republic	2
China (Hong Kong)	1
Denmark	3
Finland	1
France	15
Germany	1
Italy	2
Netherlands	5
New Zealand	1
Norway	3
Portugal	1
Russia	3
South Africa	1
Sweden	3
United Kingdom	46
United States	7

*Gender* (m 81, f 23, based on all interviewees in the series). Because of the focus on older and retired workers a male bias to the series was to be expected, given the restriction of opportunities for women in science and medicine until recently, but a notable number of important contributions to human genetics were made by women, despite the fact that they had frequently encountered major difficulties, as can be seen in the interviews.

*Age of interviewees* Most interviewees were in their 70s or 80s, born in the 1920s and 1930s, reflecting the principal aim of the series in capturing the memories of older workers still living. One consequence of this is that many interviewees had their childhood or early adult life disrupted by war, as described below.

## Major themes in human and medical genetics reflected by the interviews

Over the time span reflected in these interviews, principally the mid-1950s to mid-1980s, a succession of major themes has been at the forefront of the field, as summarised in Table 3. In scientific terms, the most notable of these is human cytogenetics, which itself provided the main stimulus for the development of medical genetics as a specific field.

**Table 3:** Major themes in human and medical genetics arising in the interview series

Human cytogenetics	
Radiation genetics	
Cancer genetics	
Human biochemical genetics	
Human population genetics	
Human molecular genetics	
Clinical genetics	

### Human cytogenetics

The focus on the beginnings of human cytogenetics in many early interviews was not surprising given that these involved the 1950s and 1960s, when the key early discoveries involving human chromosome anomalies were made and clinical applications in diagnosis (later including prenatal diagnosis) became possible. A special effort was made to interview as many as possible of those involved in the early development of this field (see Table 4).

**Table 4:** Interviews with workers in early human cytogenetics

Name	Main place of work
Bertram, Ewart/Moore, Keith^a^	London, Ontario
Berger, Roland	Paris
Bobrow, Martin	Oxford/London UK
Bochkov, Nikolai	Moscow
Brøgge, Anton/van der Hagen, CB	Oslo
de la Chapelle, Albert	Helsinki
Delhanty, Joy	London UK
Evans, H John	Edinburgh
Evans, Edward^a^	Harwell
Ferguson-Smith, Malcolm^a^	Glasgow
Fraccaro, Marco	Pavia
Gilgenkrantz, Simone	Nancy
Harnden, David^a^	Manchester
Hamerton, John	London UK/Winnipeg
Hulten, Maj	Stockholm/Birmingham
Jacobs, Patricia^a^	Edinburgh
Lee, Muriel^a^	Edinburgh
Lindsten, Jan	Stockholm
Polani, Paul	London UK
Searle, Anthony	Harwell
Sutherland, Grant	Adelaide
Turleau, Catherine	Paris
Vanden Berghe, Herman	Leuven
Zech, Lore	Stockholm

^a^Audio clip from interview also available

The earliest topic related to human cytogenetics to be captured in the interview series is the discovery of the sex chromatin body, made in 1948 and published the following year by Ewart (Mike) Bertram while working as a postgraduate student with Murray Barr in the anatomy department of University of London, Ontario (Barr and Bertram 1949). Others had previously observed the actual structure itself, but Bertram’s discovery that it was present in the brain cells only of female animals (cats) was the foundation for later research on the chromosomal basis of sex determination in mammals, including humans, which completely invalidated previous assumptions based on Drosophila. Murray Barr was no longer living to interview, but Mike Bertram and his co-interviewee and colleague Keith Moore describe the discovery:

- Mike Bertram: I had come on to the section of this one cat, drew all the measurements and then next cat the same way, and then it came to one…
- Keith Moore: Couldn’t find it!
- Mike Bertram: Couldn’t find it and didn’t tumble to it till events…
- Keith Moore: Poor staining!
- Mike Bertram: Then we started looking up my records, because I kept track of the age and the sex and the coloration and all the rest of it. So all these things now we’re recording were ‘sex female’. So we began looking back at records, and going through the series, every time it appeared in the animals they were female. Then when it didn’t appear it was male. We were attributing it to very poor staining and other things that didn’t show up. So that’s basically how we made the discovery….
- Interviewer (PSH): Can I ask, at that time what did you think this body actually was? Did you think it was anything genetic …..?
- Mike Bertram: Well at that time no…..
- Keith Moore: We thought it was RNA first and then by Feulgen stain, wasn’t that what we used, it showed up as DNA.
- PSH: So you didn’t really think that you were looking at a chromosome?
- Mike Bertram: No
- Keith Moore: No idea.
- Mike Bertram: Absolutely not!
  (Interview with Ewart (Mike) Bertram and Keith Moore, 28/10/2004). [23]^1^

This work also provided the first laboratory diagnostic application of human genetics, by Bertram’s successor as Ph.D. student to Barr, Keith Moore, who showed that the sex chromatin body could be detected by the simple technique of a buccal smear in a wide range of species, including humans.

- I was showing this and this one lady came up and said, ‘I work with ducks’, and I said well, I don’t know whether ducks have it, but the only way to find out is to take a piece of skin and check it out and see if there is any sex chromatin in it. ‘Oh I couldn’t do that with my poor ducks!’ She said ‘Can’t I just scrape the mouth of the duck?’ I said sure, you would probably get some cells and do it. Oh boy! (clicks fingers) that just clicked in my head. I went home and I started scraping. I got one of those metal spatulas, I scraped my own, I scraped my wife and my baby daughter who is now 50, and boy they showed up beautifully, because you just had to smear it on.
  Interview with Ewart (Mike) Bertram and Keith Moore, 28/10/2004. [23]

These two workers, in the joint interview made in 2004 [23], over 50 years since the initial discovery, describe their work as if it had occurred just a few weeks ago, something that can be appreciated even more graphically in the recording itself than in the transcript. Keith Moore has provided a valuable historical account of the discovery and subsequent work on sex chromatin in his book on the topic, *The Sex Chromatin* (Moore 1966). Interestingly, Moore told me that the historical chapter had to go through six drafts before everyone agreed on it!

At the time of the initial studies on human chromosomes, the precise human chromosome number was still unknown, (or rather believed erroneously to be 48, not 46. The key discovery of Tjio and Levan (1956) is not represented in the interview series since neither author was living, but a visit to Lund in 2004 allowed discussions with other contemporary workers and colleagues (see Harper 2006a, b), while the interview with Maj Hulten [10], a visiting student in Lund at the time of the 1956 discovery, also gives information on this, as does a more detailed paper by Hulten (2002). The rapid confirmation of 46 as the correct human chromosome number by Ford and Hamerton (1956) is described in the 2004 interview with John Hamerton shortly before his death [21].

The theme of the sex chromosomes and their disorders is continued in the series by the interviews with Paul Polani [01] and with Patricia Jacobs [06] and her technician Muriel Lee [12]. Polani’s observations link observations on the sex chromatin and clinical studies on Turner Syndrome with full chromosome analysis (in collaboration with Charles Ford who was no longer living to interview, though the interview with his colleague Edward Evans [15] gives considerable information on him), showing the XO karyotype now to be expected from absence of sex chromatin. Polani had previously had considerable difficulties in persuading others to accept that his sex chromatin data had consequences not only for Turner syndrome but for human sex determination generally.

- Interviewer (PSH): At what point did you manage to convince Penrose that human sex determination was different from Drosophila?
- Paul Polani: Penrose would not have it. Penrose would not have it, I have to say. He was annoyed with me. He said ‘where did you get this stupid idea? And I said ‘well yes Professor Penrose, but see, the figures would suggest that there is something.’… Well anyway, when I sent my paper into the Lancet in 1956 I had the audacity not only of suggesting that they might be XO sex but also writing that, if they were XO sex they would be unlike what happens in Drosophila….And the Lancet would not have this bit…. and said ‘No, we can’t have that sort of thing. Get him to modify it. Take it all out.’ And I said ‘No, I’m not going to take out the XO sex story’. Anyway that’s another thing.
  (Interview with Paul Polani, 12/11/2003)

Conversely, and quite independently, the work of Patricia Jacobs at the newly formed Medical Research Council ‘Group for Research on the General Effects of Radiation’, in Edinburgh, showed that a chromatin positive Klinefelter syndrome male had an XXY karyotype, while the interview with her technician Muriel Lee [12] emphasises the value of the resourceful and intelligent scientific technician in research, a group often neglected by interviewers and historians.

- Patricia Jacobs: Anyway, on we went and I looked at the Klinefelter and the preparations were really very bad, even though I had practiced. And I thought there were 47 chromosomes and there were two Xs and a Y, and remember we couldn’t even tell the Y. But I did and I couldn’t believe it. And this was not the perceived wisdom of what Klinefelter’s was in that day and age…. So I went on holiday and I asked my technician to prepare a tray of slides with the Klinefelter in it and lots of other things too, and I would come back and score them blind, and I did. I came back from my holiday and I scored them blind and I thought, well that’s funny because there seemed to be two that seemed to have 47 chromosomes, not just one as I had expected. So I said to her, I’ve got two that I really think might have 47 chromosomes, and she broke into a big grin, because she had put two from the Klinefelter’s into the tray! So I thought, well that may be true.
  (Interview with Patricia Jacobs, 13/02/2004)

Further successive developments in human cytogenetics which are reflected in the interview series include the discovery of the autosomal trisomies 13 [54] and 18 [14], described by James Crow and John Edwards, the development of chromosomal banding techniques (Lore Zech, [28]) allowing specific recognition of the individual human chromosomes, including the Y chromosome (Martin Bobrow [24]), and the cytogenetic (and later molecular) basis of fragile X syndrome, as described by Grant Sutherland [60].

Prenatal diagnosis of chromosomal disorders rapidly become an important part of human cytogenetics once the culture of amniotic fluid cells became technically feasible, and is represented in the interview series by workers such as André and Joelle Boué in Paris [43] and Malcolm Ferguson-Smith in Britain [03], among others.

### Other major themes (see Table 3)

Two themes closely involving cytogenetics that appear in the interview series are radiation genetics and cancer genetics.

*Radiation genetics* was a particularly prominent area of human genetics during the 1960s and 1970s, with concerns over potential harmful effects of radiation heightened by atomic testing in the atmosphere and the dangers of the cold war, as well as by increasing medical use. These concerns led to important institutional and funding support for cytogenetics and for human genetics generally in numerous countries, including Scandinavia, Italy and Germany. In Britain, the two principal Medical Research Council units involved with human cytogenetics in Edinburgh and Harwell were specifically designated for radiation research, though fortunately this did not stop them from straying well beyond their official remit. Both are prominently represented in the interview series and include Patricia Jacobs [06], David Harnden [08] and H John Evans from Edinburgh [04] and Mary Lyon [18], Anthony Searle [19] and Edward Evans [15] from Harwell.

Perhaps the most extreme situation of human genetics linked to radiation research is seen in Russia, where the three successive directors of the renewed Moscow Medical Genetics Institute—Nikolai Bochkov [46], Vladimir Ivanov and Yevgeny Ginter [47], as well as Zhores Medvedev [58], who was expelled from the Soviet Union—were all based originally in radiation genetics research under Nikolai Timofeef-Resovsky, one of the few survivors of the repression of genetics under the influence of T. D. Lysenko during the years of Stalin and Kruschchev. More recent problems related to radiation are illustrated by the interview with Minsk pathologist and geneticist Gordon Laziuk [45], relating his experiences following the 1986 Chernobyl disaster. Although he was ideally placed to examine the genetic effects of radiation exposure, having maintained a register of congenital malformations covering the affected area for some years, he (and others) initially met with nothing but denial from the authorities, even the radiation doses involved being concealed until reported from abroad. Continuation of his work eventually led to him being dismissed from his post.

*Cancer genetics* has been part of human cytogenetics since techniques permitted a meaningful analysis of human chromosomes, indeed even before this—study of chromosomes in tumours was the key factor stimulating Levan and Tjio to determine unambiguously the normal human chromosome number. After Nowell and Hungerford (1960) had first shown a specific somatic chromosome abnormality in chronic myeloid leukaemia, the field of cancer cytogenetics was advanced further by Lore Zech’s discovery of banding in human chromosomes [28], (Caspersson et al. 1970), and the increasing volume of research internationally brought together by the Cancer Chromosome Database of Felix Mitelman [92]; both are represented in the interview series, as are a number of other workers in the field (see Table 5).

**Table 5:** Principal themes of interview series cancer and genetics

Roland Berger	
Albert de la Chapelle	
H John Evans	
David Harnden	
Henry Harris	
Felix Mitelman	
Giovanni Romeo	
Julian Sampson	
Herman Vanden Berghe	
Friedrich Vogel	
Lore Zech	

*Biochemical genetics* was an important element of human genetics from the outset, indeed from the time of Garrod’s original discovery of inborn errors of metabolism at the beginning of the 20th Century, though not always such an integral part of it as has been cytogenetics. Among those interviewees involved with research on inherited metabolic disorders some, such as Jean Frézal and Charles Scriver, were closely identified with broader medical genetics, while others were more based in biochemistry, the former group being those mainly represented in the interview series (Table 6). An important group of workers involved in human biochemical genetics were those engaged in human gene mapping, especially in the decades 1970–1990, before molecular genetic approaches became dominant. This group in turn overlapped with those in population and statistical genetics (Table 7).

**Table 6:** Main themes represented in the interview series: biochemical genetics

Jean Frézal	Inherited metabolic disorders; human gene mapping
David Hopkinson	Human biochemical genetic variation
Jean-Claude Kaplan	Inherited metabolic disorders
Sue Povey	Biochemical polymorphisms; human gene mapping
Charles Scriver	Inherited metabolic disorders
Hans Eiberg	Human gene mapping
Hans Galjaard	Inherited metabolic disorders

**Table 7:** Principal themes of interview series: human population genetics

R J (Sam) Berry
Walter Bodmer
Michael Conneally
James Crow
Anthony Edwards
John Edwards
Josué Feingold
Yevgeny Ginter
Trefor Jenkins
Newton Morton
Derek Roberts

*Human molecular genetics* (Table 8) was a late arrival in comparison with the other laboratory genetics approaches mentioned so far, and most of its pioneers originated from a biochemistry or basic molecular biology background rather than from cytogenetics Indeed, there are fundamental differences between the microscopy approach of cytogenetics and these other technologies that have persisted until very recently, though most research workers in human genetics adapted rapidly to the new molecular era.

**Table 8:** Interviewees involved with the beginnings of human molecular genetics

Walter Bodmer	Oxford, London, Stanford
Gillian Bates	London
Egbert Bakker	Leiden
Russell Snell	Cardiff, Auckland
Jean-Louis Mandel	Strasbourg
Jean-Claude Kaplan	Paris
Alec Jeffreys	Leicester
Grant Sutherland	Melbourne
Nick Hastie	Edinburgh
Robert Williamson	London, Melbourne
GertJan van Ommen	Leiden
Kay Davies	London, Oxford
Peter Goodfellow	London, Cambridge
James Gusella	Boston
Marcia MacDonald	Boston
Allan Tobin	Los Angeles

At the time when I began the interview series, most of the workers in human molecular genetics were still active and relatively young, so I did not initially regard them as a priority group for interviewing. But quite soon I realised that the field of human molecular genetics, or at least its beginnings, was becoming part of the history of the field, and that the pioneer workers were starting to retire. I therefore shifted my emphasis in the later part of the interview series to try to cover human molecular genetics, though I realised that I could only see a small fraction of those involved. I tried to focus especially on those involved in disease-related molecular research and in applications to genetic testing for inherited disorders, rather than trying to interview scientists involved in more fundamental research, who I felt would be more likely to be interviewed by others more suitable than myself for this. I have been proved correct by the appearance of some other interview collections focused on basic science, such as that of Gitschier (2010). In particular I did not attempt to cover the Human Genome Project, which from the outset has attracted the attention of historians and journalists, and for which ongoing projects are being supported by bodies such as Wellcome Trust and Cold Spring Harbor Laboratory.

A particularly striking example of the power of molecular approaches to human—and much wider than human—genetics is the discovery of DNA fingerprinting, as told here by its discoverer Alec Jeffreys [75]:

- So just to check this idea before taking this little shared core motif and using that to go into a genomic library, the obvious experiment was simply to take a repeated core probe and hybridize it to total genomic DNA to check whether it picked up multiple variable minisatellites. That was the key, almost accidental, experiment that triggered the entire field of human DNA identification. On the autoradiograph that we got was a set of fuzzy bar-code-like patterns coming out from the three individuals that we had on that Southern Blot. …We could tell those three people apart, and you could see how the child’s fingerprint was a composite of mum and dad’s, so we could immediately see biological identification using DNA and we could see establishing family relationships. All of this was gained purely by accident on this first Southern blot. We had a whole lot of non-human species on the blot too. So there was a mouse, a rat, a cow, a seal, a lemur, a baboon, tobacco DNA, and just about everything came up with what looked like a DNA fingerprint. It was an extraordinary moment and I think the penny dropped within seconds from that first autoradiograph coming out of the developing tank. I think my first reaction was what the hell is going on here, what a mess, and then the penny dropped. Here was DNA-based biological identification, family relationships and then all of the non-human applications, from dog paternity disputes to conservation biology, biodiversity monitoring, it was all there. It was a very exciting moment. I’d never ever planned to come up with a technology for identification; we just found it.

As with many discoveries in genetics, the interval between basic science discovery and practical applications was a short one.

- The following sequence of events meant that I was now embarking on what I call the great detour of my academic life, which was to go charging off into the world of forensic and legal medicine. So the sequence of events was that we published this in Nature, and in the paper we speculated on biological identification, though for patenting reasons we said little about the animal identification. That article was picked up by Andrew Veitch, a science correspondent with The Guardian, he wrote a lovely little piece on it that was read by a lawyer in London who represented a family involved in a very tricky immigration dispute. They’d been through all the blood group testing that basically failed to convince anybody of anything so she then wrote to me and said, look I’ve heard about this new fangled DNA stuff, could you possibly help with this family? And I thought ok, right this is, we’d done a lot more work; fuzzy blobby bands had turned into something quite pretty and highly informative, so we thought ok this is crunch time now, you cannot possibly say no to this woman. So that was our first case, which had a successful resolution, a young lad facing deportation reunited permanently with his family. It was a good news story, a great story. So that was the trigger and as soon as publicity came out on this case there was an avalanche of enquiries—I’d no idea of how many people were trapped in immigration disputes, they all wanted DNA testing. So that case was done in April 1985, and I think it was in June that the immigration tribunal dropped the case against this boy. By the summer of 1985 we’d taken on the first paternity dispute anywhere, to my knowledge, and that then opened another flood gate, and then life went completely mad.
  (Interview with Alec Jeffreys, 16/02/2010).

One reason for urgency in interviewing human molecular geneticists is the relative fragility of much of their associated historical material. Most of the older scientists that I had been interviewing had large collections of correspondence and other physical items such as books and papers, which could be salvaged for archiving if this was arranged while the person was still living. Indeed, such material formed the basis for other initiatives of the Genetics and Medicine Historical Network, as can be seen on its website (http://www.genmedhist.org) The interviews thus stimulated the cataloguing and archiving of a series of extensive sets of personal scientific records, while many books were donated by interviewees, making up the great majority of the Human Genetics Historical Library, now amounting to well over 3000 books (Harper and Pierce 2010).

By contrast, present day scientists, including most of those who founded human molecular genetics, have almost exclusively used email for their correspondence, while most other documents are also electronic; primary research output is likewise mostly non-visual, in contrast to the images previously produced by cytogeneticists. Although in theory it should be easier to preserve and archive this electronic material, it is also extremely easy to destroy it, in particular correspondence. It requires an awareness of the importance of saving this material if it is not to be irrevocably lost.

Table 8 lists those people interviewed who have played a prominent role in human molecular genetics; for the UK a ‘witness seminar’, ‘Clinical Molecular Genetics in the UK’ (Jones and Tansey 2014) records a group discussion on the topic.

## Medical genetics

Medical genetics as a specific scientific and medical discipline, especially clinical genetics as that part of it involving patient services and clinically related research, largely followed the developments in more basic human genetics described above, as they became applicable to medical practice and created the need for medically trained workers who could handle the diagnostic and other clinical aspects of these new discoveries. Throughout the 1960s and 1970s, both in North America and in Europe, new medical genetics centres were created, some academic, others health-service funded, which could satisfy clinical demand for genetic diagnosis and genetic counselling; this also provided opportunities for research using the increasingly extensive medical data that were becoming available.

Among major advances in medical genetics that were captured in the interview series through interviews with some of the key workers involved were the molecular basis, prenatal diagnosis and prevention of the thalassaemias (David Weatherall [30], Bernadette Modell [70]); prevention of neural tube defects (Rodney Harris [59], K Michael Laurence [13]), and Duchenne muscular dystrophy (Bert Bakker [77], GertJan van Ommen [78], Kay Davies [80]). A number of workers involved with isolation of the Huntington’s disease gene, a special interest of the author, were also interviewed, on both sides of the Atlantic, including James Gusella and Marcia MacDonald [82], Gillian Bates [57], Michael Conneally [22], Russell Snell [83] and Alan Tobin [52].

Many of these medical geneticists were at the interface of basic research and application, an essential though often undervalued role. Bernadette Modell’s work on thalassaemias provides an example of this; after setting up a thalassaemia clinic in London and realising that many couples with an affected child requested abortion rather than risk the recurrence of the disorder, she collaborated both with gynaecologists developing techniques of fetal blood sampling and subsequently first trimester chorion villus sampling, and also with molecular geneticists. She describes their first attempt at using this for early prenatal diagnosis:

- So now among these families, there was one, and actually he was an Imam, a Mullah, and they had a child with thalassaemia. They were one of these families that came down to London for prenatal diagnosis, mid-trimester prenatal diagnosis and they came and the fetus was affected and they had a mid-trimester abortion and they felt obviously terrible, as one does. And she got pregnant again. A…… had told them about our efforts for first trimester diagnosis, so they came down to London to see us, and said they wanted us to try and I said to him, I asked him whether he felt that by doing this they would be contributing to developing techniques that were acceptable to their community and he said yes, they also thought that. So that was our first real case and we did it here at the Temperance Hospital, in the same operating theatre where we had been doing the research. This was interesting because sometimes you are so focused on what you are doing that you don’t realise what’s going on around you. The theatre sister burst into tears. She said, it was wonderful seeing something good come out of all of this. There are so many good people and they become so involved in the objective that you are trying to reach and sometimes you don’t see them because they are behind you, not in front. Now that foetus was affected too and she had an early abortion. And when A…… went to see the family later in Bradford, she said “It’s wonderful. I can’t tell you the difference”. And they got pregnant again and it was alright the next time. When we finally published on the first series, in the acknowledgements, I felt I had to acknowledge the 22 brave ladies who had made all these decisions which led us on and on to getting something which was better.
  (Interview with Bernadette Modell 14/12/07)

### Clinical genetics

While many of those in human genetics research had a medical background, the increasing medical applications, the need for diagnosis of previously undefined genetic disorders and for genetic counselling resulted in the emergence of a defined specialty of medical genetics as part of health services. This formed a broad grouping of clinicians whose origins were in other fields, such as paediatrics or internal medicine, but who progressively became fully involved as clinicians in medical genetics. Numerically, these are prominent in the interview series, a bias resulting in part from the author himself being a clinical geneticist. This group is, however, especially important since it contains many individuals who were the key founders of medical genetics in a number of countries and regions, who were responsible not only for important research and academic developments in the field, but for the introduction of genetic services and their integration into the overall medical structures of their specific countries.

For the UK an attempt was made to cover all the principal founders of clinical genetics still living, along with others who had main notable contributions in the early years of development of the field (Table 9). For most countries of continental Europe this was not possible, so a few key individuals were (somewhat arbitrarily) chosen, and encouragement given to others to ensure that a wider range of workers in each country should be interviewed. The European Society of Human Genetics (ESHG) and a number of national human genetics societies have now begun to undertake this.

**Table 9:** Interviews with UK clinical geneticists

Name	Main place of work
Baraitser, Michael	London
Berry, Caroline	London
Bobrow, Martin	London/Cambridge
Burn, John	Newcastle
Clarke, Angus	Newcastle; Cardiff
Donnai, Dian	Manchester
Edwards, John	Birmingham (UK); Oxford
Emery, Alan	Manchester/Edinburgh
Farndon, Peter	Birmingham (UK)
Fraser, George	Oxford
Harper, Peter (interviewer Angus Clarke)	Cardiff
Harris, Rodney	Manchester
Hughes, Helen	Toronto; Cardiff
Johnston, Alan	Aberdeen
Laxova, Renata	Brno/London/Madison
Nevin, Norman	Belfast
Pembrey, Marcus	London
Sampson, Julian	Cardiff

The interviews reflect an increasing trend from research to service development, but also a strong tradition of combining the two, and in particular to make maximal use of the research opportunities provided by patients often seen primarily for service reasons. They also show how to a large extent genetics services (including prenatal diagnosis) were developed initially using research funding, but later were able to create their own health service support in terms of funds and specific staff, which in turn provided foundations for further research. A number of the interviews also document the initial opposition to prenatal diagnosis, as in France, Belgium and Norway, something largely overcome by medical geneticists showing that it was being undertaken cautiously and responsibly.

Since most of these interviews were made, a ‘Witness Seminar’ on the origins and development of clinical genetics in Britain (Harper et al. 2010) has provided a permanent record of a group discussion on this theme, many of the participants having already contributed recorded interviews to the present series. It should also be noted that over the past 20 years in Britain and a longer period in America, a specific category of non-medical genetic counsellors has developed; it is to be hoped that a recorded interview series will be created that specifically covers the early workers responsible for this, since they are under-represented in the present interviews.

## Interviewees since deceased

One of the primary aims of the interview series was to capture and preserve the memories of workers who, on account of their age, might be expected not to live much longer. The value of this policy has already been shown since at least 21 of the interviewees are already deceased over subsequent years, as indicated in Table 1.

In addition, a number of other workers, for whom interview was already planned, died before this could be achieved, including Margaretha Mikkelsen (Copenhagen), E A (Tony) Murphy (Baltimore/Barcelona), Eeva Therman (Helsinki/Madison) and Robin Winter (London).

This inevitable mortality also emphasises the need for a continuous process of interviewing, especially important in such a rapidly changing field as human genetics.

## Teachers and mentors; the older generation

One of the few deliberately constant questions in the interviews was regarding the person (or persons) most influential in an interviewee’s career and life, a question which provided detail on a number of important figures in genetics from a previous generation, mostly already deceased. Considerable information on such early pioneers commonly arose earlier in the interviews in relation to a person’s training and early career.

Since most interviewees were already in their 60s to 80s, many of their mentors had been deceased for a considerable time, with careers reaching back before World War 2 into the 1930s, before medical or human genetics had become a differentiated field from more general genetics. Table 10 lists some of these individuals, about whom a significant amount of information was recounted in the interviews, and which helps to extend our knowledge of the origins and development of the field back a generation further. Some names recurred frequently, such as Lionel Penrose of the London Galton Laboratory, indicating his major influence on the development of human genetics internationally.

**Table 10:** Individuals, mostly already deceased, for whom information is provided by living interviewees

Individual	Interviewee
Carter, Cedric	Michael Baraitser, Michael Laurence, Norman Nevin, Marcus Pembrey
Barr, Murray	Mike Bertram/Keith Moore
Brock, David	Alan Emery
Caspersson, Torbjo̎rn	Jan Lindsten, Lore Zech
Cavalli-Sforza, Luca	Walter Bodmer, Anthony Edwards
Clarke, Cyril	Peter Harper, David Weatherall, Marcus Pembrey
Cook, Peter	Sue Povey
Court Brown, Michael	John Evans, David Harnden, Pat Jacobs
Dent, Charles	Charles Scriver
Fisher, RA	Walter Bodmer, Anthony Edwards, Sam Berry, Mary Lyon
Ford, Charles	John Evans, Ted Evans, John Hamerton, David Harnden, Paul Polani
Ford, EB	Walter Bodmer
Gru̎neberg, Hans	Caroline Berry, Sam Berry, Anthony Searle
Haldane, JBS	John Evans, John Hamerton, Newton Morton, Robert Williamson
Harris, Harry	Sue Povey, Charles Scriver
Hogben, Lancelot	John Edwards
McKusick, Victor	Alan Emery, Malcolm Ferguson-Smith, Peter Harper, Alan Johnston, Giovanni Romeo
Mohr, Otto Lous	Kåre Berg, Jan Mohr
Nachtsheim, Hans	Friedrich Vogel
Neel, James	Derek Roberts
Patau, Klaus	James Crow
Penrose, Lionel	Marco Fraccaro, Joy Delhanty, Renata Laxova, Ursula Mittwoch, George Fraser, Paul Polani, Herman van den Berghe
Polani, Paul	Caroline Berry, Martin Bobrow, John Hamerton, Marcus Pembrey
Renwick, James	John Edwards, Anthony Edwards, Malcolm Ferguson-Smith, Newton Morton
Roberts, John Fraser	Caroline Berry, Paul Polani, Marcus Pembrey
Robson, Elizabeth	Sue Povey
Steinberg, Arthur	Trefor Jenkins
Stevenson, Alan	Martin Bobrow, John Edwards, Derek Roberts, Marco Fraccaro, George Fraser, Norman Nevin
Timofeef-Resovsky, Nikolai	Zhores Medvedev, Yevgeny Ginter, Nikolai Bochkov
Winter, Robin	Michael Baraitser, Dian Donnai
Wright, Sewall	James Crow

## Personal aspects

These were not enquired about directly in the interview, though the use of a biographical approach in most cases meant that they were naturally prominent in accounts of early childhood and family background. Interviewees were told specifically that any areas that they considered sensitive or inappropriate for the edited Web version, could be omitted or removed, occasionally they were postponed for informal discussion after the recording had been completed.

In fact very few such situations occurred, and during the editing process very little was usually changed or removed by participants, though some required considerable persuasion not to try to ‘improve’ their grammar and style in the transcript! Much detail of a personal nature was raised spontaneously, adding considerably to the vividness and authenticity of the interviews. This is especially apparent when listening to the recordings themselves, and it is greatly to be hoped that ‘audio clips’ will in future be able to be made for all interviews, comparable to the 10 already constructed and available on the Genmedhist website.

A factor contributing to the willingness of most interviewees to include material that others might consider ‘sensitive’ may have been that the events involved were often several decades ago, that the author as interviewer had not been involved with the work, and was often from a different country. In some cases the interview was the first occasion that the interviewee had spoken about the particular topic. This was notably the case in relation to the traumatic years of younger life and childhood encountered by many individuals originally from continental Europe, whose lives had been disrupted by World War 2 and other political catastrophes, something on which I give a brief separate note here, but which deserves a fuller account (see Table 11).

**Table 11:** Interviewees affected by World War 2 and other major political problems

Name	Nature of problem
Michael Baraitser	South African apartheid
Nikolai Bochkov	Childhood in wartime Russia
Martin Bobrow	South African apartheid
Walter Bodmer	Parents refugees from Nazi Germany
George Fraser	Refugee from Nazi Germany (with parents)
Trefor Jenkins	South African apartheid
Jean-Claude Kaplan	Clandestine life in occupied wartime France
Michael Laurence	Childhood refugee from Nazi Germany
Renata Laxova	Refugee from communist Czechoslovakia
Gordon Laziuk	Dismissed from post because of work on Chernobyl nuclear disaster
Milan Macek	Communism in Czechoslovakia
Zhores Medvedev	Expelled from communist Soviet Union
Felix Mitelman	Childhood refugee to Sweden (with parents) from post-war Poland
Ursula Mittwoch	Childhood refugee from Nazi Germany. Interned as ‘enemy alien’
Paul Polani	Refugee from fascist Italy. Interned as ‘enemy alien’
Derek Roberts	Severely wounded in World War 2
Anthony Searle	Prisoner of war in Japanese-occupied Singapore
Friedrich Vogel	Wounded and prisoner of war on Russian front
Loe Went	Resistance activities in wartime Netherlands

## War and persecution

Sixty years on from World War 2 it is becoming all too easy to forget the immense disruption that it caused, both to individual lives and to institutions and research overall, especially across Europe, though actual loss of life for scientists and medical doctors was perhaps less than in the indiscriminate carnage of the First World War; J. B. S. Haldane, himself wounded, had to complete his 1915 paper describing the first genetic linkage in mammals (Haldane et al. 1915) in the trenches, his co-author having been killed in battle. His biographer Ronald Clark notes:

- He was, as he later was to boast, the only officer to complete a scientific paper from a forward position of the Black Watch. This was the final result of the mice experiments, started before the war with Naomi [his sister] and AD Sprunt who had recently been killed in action. JBS had written to William Bateson, one of the leading British geneticists and Director of the John Innes Horticultural Institution at which Haldane was later to work, and told him of the mice experiments, characteristically adding: “if I am killed could you kindly give my sister help if she wants it.” Shortly afterwards, working in the trenches, JBS finished the paper.
  (Clark 1968).

Only the older interviewees in this series were combatants; among these, Derek Roberts [02] lost an arm; Friedrich Vogel [05], as a teenager in the German army, was wounded and imprisoned on the Russian front; while Anthony Searle [19] was a Japanese prisoner of war in Singapore.

Others affected were children at the time. Jean-Claude Kaplan [40], as a Jew in Vichy France, lived under a changed identity, while Nikolai Bochkov [46] had, at the age of 10, to work on the local collective farm with his older brother, since all the adults in the family were in the army and some dead.

The decade of fascism before the war produced a stream of refugees from continental Europe to Britain and, to a lesser extent, America. Those in the interview series were too young to be among the established scientists, mostly Jewish, who were found posts in Britain, and who included such eminent geneticists as Hans Grueneberg, Hans Kalmus and Charlotte Auerbach. Again, most were children, fleeing with (or without) their families in the years immediately before the war. Those in the present interview series included George Fraser [32], Michael Laurence [13], Ursula Mittwoch [07] and Renata Laxova [55], who has described her experiences in her autobiography (Laxova 2001). A vivid account has also been given elsewhere by Arno Motulsky (http://www.nytimes.com/2008/04/29/science) of his attempts to reach America from Nazi Germany.

An ironic consequence for many of these refugees was to be interned as ‘enemy aliens’ in the Isle of Man internment camp, often alongside British fascists and Nazi supporters. A detailed account of this little remembered camp is given in the book *Island of Barbed Wire*, (Chappell 1984), while Max Perutz’s account of his experiences, ‘Enemy Alien’, told with wry humour, is given in his collected essays (Perutz 1998). Among interviewees in this series, Ursula Mittwoch [07], who had come with her family from Berlin as a schoolgirl in early 1939, soon found herself interned on the Isle of Man, something that later prevented her going to University, but not from developing a distinguished academic career.

- UM: I left Germany at the age of 15; actually I had to leave school at 14, and so there was a bit of an interregnum between leaving school and coming to England, and I finished school, …. we came to England in the Spring of 1939, that was about 6 months or so before war started, world war 2, and then, well when my parents were looking for somewhere to live, my sisters and I were sent to boarding school in Brighton for a year, …. and then, yes was it the fall of France, the invasion of Holland and Belgium? 1940. Well that was a cue to intern certain aliens, certain foreigners. …. Now my parents had been exempt from internment because at the beginning of the war they went before a tribunal and they were exempt. Well, I hadn’t been before a tribunal because I wasn’t 16 and then of course I was 16 just before and so that was bad luck.
- PSH. (interviewer): Oh dear.
- UM. So I went to the Isle of Man and my younger sister was jealous, she wanted to, she liked to travel but she had to stay at home and I went off for about 9 weeks and then, you know, they brought this sort of category of, I don’t know, children, teenagers or whatever and they brought them back. But it didn’t do my schooling any good.
  (Interview with Ursula Mittwoch, 02/02/2004)

Refugees from Nazi Germany were not the only ones affected by internment. Paul Polani [01] had come from Italy to Britain for postgraduate study immediately before the outbreak of war, but, since this was now impossible, became a ship’s doctor. When Italy entered the war he was at once interned as an ‘enemy alien’, narrowly missing transportation to Canada on the ship *Arandora Star*, which was torpedoed and sunk with heavy loss of life; Polani had been posted to London shortly before this to do a medical locum post, which in fact lasted for the entire war and provided the starting point for his distinguished career at Guy’s Hospital and as a founder of British medical genetics.

The end of the war did not mean the end of disruption of lives and careers for many geneticists. Eastern Europe in particular had now come under Soviet domination and Lysenkoist doctrines became mandatory. Scientists became skilled at disguising genetic research, particularly in the radiation biology and microbial genetics fields; medical workers classed their research as part of paediatrics or pathology (see Chapter 16 in Harper 2008). Renata Laxova describes the situation for Czechoslovakia in her interview [55] and her autobiography (Laxova 2001), as well as the memorable description of her welcome by the Penroses when she and her family appeared on their doorstep after fleeing Czechoslovakia following the 1968 Russian invasion.

In the Soviet Union itself, the successive catastrophes for genetics, starting in the 1930s and continuing for almost 50 years, have been well documented in a number of books by Russian geneticists, such as Medvedev (1969), Soyfer (1994) and Berg (1988), but the effects of the persecution on human and medical genetics have not yet been fully analysed. The interviews with Zhores Medvedev [58], Yevgeny Ginter [47] and Nikolai Bochkov [46] in this series give a glimpse of the problems during the Lysenko era; even after its end, any criticism of this period was firmly suppressed, so that most of its chroniclers (including Medvedev) were expelled from Russia.

Among other countries that were a notable source for future medical geneticists as migrants, if not actual refugees, to Britain was South Africa during the apartheid period. In the interview series both Martin Bobrow [24] and Michael Baraitser [33] left the country soon after finishing their medical training on account of the political situation. Conversely, it should be noted that Trefor Jenkins [69], born in Britain, moved permanently to South Africa and played a distinguished part in the resistance to apartheid, as can be seen in the interview, in addition to developing the country’s foremost human genetics unit.

## Discussion

This series of 100 recorded interviews with human and medical geneticists (104 individual interviewees) described here is the most extensive undertaken so far, and the only one for which almost all edited interview transcripts are available on the World Wide Web. The systematic archiving of all sound recordings, correspondence and other background material associated with the project (Cardiff University Special Collections and Archives 609, Prof. Peter Harper archive) should be of additional value to future scholars wishing to analyse the origins and development of human and medical genetics internationally over the second half of the 20th Century.

It should be noted, though, that the material presented here and elsewhere has significant limitations, particularly, perhaps for the professional historian (which the author is not). The series is biased in terms of geography, notably an over-representation of UK and deficit of American workers, as explained earlier. The principal areas of human and medical genetics covered are likewise biased, the coverage of early human cytogeneticists being relatively complete as part of work for a book on this by the author (Harper 2006a, b), while the emphasis on early UK clinical geneticists is in part explained by the author being one of this group.

From the outset, the interview programme had two particular aims: firstly, to preserve the oral history represented by memories of those involved in the early years of the field, especially those (the great majority) whose advanced age meant that urgent action was required if these were not to be irretrievably lost. A second aim was to encourage other geneticists and professional historians to undertake comparable interviews; especially of younger workers and other areas of human genetics relatively neglected in the present series, as well as those countries (the majority) not adequately covered. Thus, the present series represents only a start, perhaps inevitably so given its dependence on the time and effort of a single worker (the author). Initiatives are now underway, coordinated by the European Society of Human Genetics (ESHG) and by a number of national human genetics societies, to interview as many as possible of the key workers in specific countries across Europe, and hopefully the rest of the world. In North America the programme previously started, comparable in nature to that described here, has unfortunately been suspended, but 30 transcripts are available on the website (http://ohhgp.pendari.com/collection), while basic scientists are well represented in the Cold Spring Harbor archive (library.cshl.edu/archives) and in the valuable collection of recorded interviews by Gitschier (2010). It is also encouraging that an increasing number of professional historians of science and medicine are turning to human and medical genetics as an area deserving of detailed historical study, and finding material of rich interest that most had been unaware of at the start of the present interview series.

It is likely that considerably more interviews with human geneticists have actually been carried out already than is generally recognised at present, and that many are archived as ‘supplementary material’ to theses and other publications, or not securely archived at all. An initiative to identify, catalogue and where necessary translate such existing interviews would be of considerable value, and might well provide information on deceased individuals where no other interview material exists. Much of this material would be suitable (with appropriate privacy restrictions) to place on the Web.

In addition to interviews with individual workers, the ‘Witness Seminar’ offers a valuable and complementary approach to preserving the oral history of human genetics. In Britain this has been strongly developed across a wide range of medical fields, with recorded discussions involving a small group of founders in a particular area, and transcripts with detailed annotations being made available both in book form and on the Web. (The lack of annotations in the present series is something that the author particularly regrets, and this might still be remedied in future, should assistance become available). Fortunately, several such Witness Seminars now cover different areas of human and medical genetics, recent examples including *Clinical Genetics in the UK* (Harper et al. 2010); *Clinical Cancer Genetics* (Jones and Tansey 2013) and *Clinical Molecular Genetics* (Jones and Tansey 2014).

In conclusion, the transcripts and other material from this series of 100 interviews should provide a valuable resource for more detailed study of the beginnings and development of the field of human genetics, and in particular the applications to medicine of the advances, both in the practice of medical genetics and increasingly, medicine as a whole. The interviews are complementary to other approaches to oral history, and to written records of the field such as correspondence and books, and all are important if the history of human and medical genetics is to be fully documented.

## Footnotes

1 [NB: The use of numbers in square brackets [–] indicates the chronological sequence (approximate) of the interviews].
